# Evaluation of opt-in HIV testing in the construction workplace using the socioecological framework

**DOI:** 10.1093/eurpub/ckac129.473

**Published:** 2022-10-25

**Authors:** H Blake, S Somerset, W Jones, C Evans, C Cirelli, D Mbang

**Affiliations:** 1 School of Health Sciences, University of Nottingham, Nottingham, UK; 2 NIHR Nottingham Biomedical Research Centre, University of Nottingham, Nottingham, UK; 3 School of Medicine, University of Nottingham, Nottingham, UK

## Abstract

**Background:**

Late diagnosis of HIV remains a challenge and the construction workforce has several risk factors for HIV. In the Test@Work programme, we delivered HIV tests embedded within a general health check to construction workers, with high uptake and acceptability. Here, we report the experiences of construction managers and health professionals involved in Test@Work and explore the suitability of construction worksites as a venue for opt-in HIV testing.

**Methods:**

Qualitative interviews (n = 24) were conducted with construction managers who facilitated events (n = 13), and HIV/health check delivery partners (n = 11) at 21 Test@Work events held on construction sites. Interviews explored experiences of events and views towards workplace HIV testing. Event exit questionnaires (n = 107) completed by delivery partners provided qualitative data identifying facilitators and barriers to effective delivery. Thematic analysis identified themes that were mapped against a socioecological framework (individual, interpersonal, organisational, industry, public health).

**Results:**

Delivery partners reported high engagement of construction workers with workplace HIV testing, peer-to-peer encouragement for uptake, and value for accessibility of onsite testing. HIV professionals valued the opportunity to reach an untested population, many of whom had a poor understanding of their exposure to HIV risk. Managers valued the opportunity to offer workplace health checks to employees but some identified challenges with event planning, or provision of private facilities.

**Conclusions:**

The construction sector is complex with a largely male workforce. Providing worksite HIV testing and education to an untested population who have poor knowledge about HIV risk helped to normalise testing, increase uptake, and reduce HIV stigma. However, there are practical barriers to testing in the construction environment. This has global implications for delivery of HIV testing in construction workplace settings.

**Key messages:**

• Delivering workplace HIV testing as part of a general health check helps to normalise HIV testing and reduce HIV-related stigma.

• Workplace testing is convenient, accessible and reaches populations at risk for HIV, but there are some barriers to implementation of rapid tests on construction sites.

